# Common Ancestry Is a Poor Predictor of Competitive Traits in Freshwater Green Algae

**DOI:** 10.1371/journal.pone.0137085

**Published:** 2015-09-08

**Authors:** Anita Narwani, Markos A. Alexandrou, James Herrin, Alaina Vouaux, Charles Zhou, Todd H. Oakley, Bradley J. Cardinale

**Affiliations:** 1 BU-G11 Überlandstrasse 133, Department of Aquatic Ecology, Eawag (Swiss Federal Institute of Aquatic Science and Technology), 8600, Dübendorf, Switzerland; 2 4101 Life Sciences Building, UCEN Road, Department of Ecology, Evolution and Marine Biology, University of California Santa Barbara, Santa Barbara, CA, 93106, United States of America; 3 1556 Dana Building, 440 Church Street, School of Natural Resources and Environment, University of Michigan, Ann Arbor, MI, 48109–1041, United States of America; National Taiwan University, TAIWAN

## Abstract

Phytoplankton species traits have been used to successfully predict the outcome of competition, but these traits are notoriously laborious to measure. If these traits display a phylogenetic signal, phylogenetic distance (PD) can be used as a proxy for trait variation. We provide the first investigation of the degree of phylogenetic signal in traits related to competition in freshwater green phytoplankton. We measured 17 traits related to competition and tested whether they displayed a phylogenetic signal across a molecular phylogeny of 59 species of green algae. We also assessed the fit of five models of trait evolution to trait variation across the phylogeny. There was no significant phylogenetic signal for 13 out of 17 ecological traits. For 7 traits, a non-phylogenetic model provided the best fit. For another 7 traits, a phylogenetic model was selected, but parameter values indicated that trait variation evolved recently, diminishing the importance of common ancestry. This study suggests that traits related to competition in freshwater green algae are not generally well-predicted by patterns of common ancestry. We discuss the mechanisms by which the link between phylogenetic distance and phenotypic differentiation may be broken.

## Introduction

Phylogenetics has recently been integrated with community ecology to ask whether common ancestry between species, measured as their phylogenetic distance, can help explain phenomena ranging from community assembly [1–4], species invasions [5], priority effects [6], and biodiversity-ecosystem functioning relationships [7–10]. The desire to incorporate phylogenetics into community ecology has been based on many factors. First, the increased accessibility of genetic sequence data has made the construction of molecular phylogenies more feasible. Second, the availability of molecular phylogenies has made it possible to rigorously test some old and popular hypotheses, many originated by Darwin, about how species' relatedness should impact the strength of species interactions [3, 4, 11, 12]. Third, the possibility that phylogenetic relationships among species could be used to predict processes and patterns in community ecology would mean that phylogenetic information could be useful in making conservation and management decisions aimed at the protection of biodiversity, ecosystem functioning and ecosystem services [9, 10, 13, 14].

All investigations of the importance of phylogenetic distance as a predictor of ecological interactions and community assembly ultimately rely on the assumption that the traits determining species' ecologies display a phylogenetic signal [15]. When ecological traits do display a phylogenetic signal, patterns of common ancestry can be used as reasonable proxies for species' trait variability. The expectation that closely related species should be more phenotypically similar than distantly related species is intuitive, and it is also a pattern that is predicted by certain null models of trait evolution. Under such models, species accumulate greater phenotypic differences from one another, the longer the time they have had to diverge from one another [16]. However, a number of evolutionary processes can reduce or eliminate phylogenetic signal [17, 18], and recent reviews have shown that the presence of a phylogenetic signal in traits may not be as common as is widely presumed in studies of community phylogenetics [15, 18–21]. So, while phylogenetic approaches may offer great promise in community ecology, it is imperative that we first test the fundamental assumption that traits display a phylogenetic signal in individual systems in which we hope to use this assumption.

In this paper we investigate whether traits that are thought to predict the outcome of competition in freshwater algae display a phylogenetic signal. Algae have long been used as a model system in which to study community assembly and the ecological processes governing biodiversity [22–29]. This interest is partly due to the fact that one can find a great diversity of algal species coexisting in relatively homogenous environments, despite the fact that they compete for a relatively small number of limiting resources (Hutchinson’s famous “paradox of the plankton” [30]). Previous work, however, indicates that phylogenetic distance among freshwater algae is not a good predictor of the strength of species interactions [31], the probability two species will coexist [3], or how polycultures influence ecosystem functions like biomass production [10]. These previous studies showed that the phylogenetic distances among species pairs do not predict the outcome of community assembly and species interactions for this group of algae. Such prior results suggest that traits related to competition and coexistence may not show a phylogenetic signal, but this assumption has never been tested. This is the first direct investigation of how species’ competitive abilities for inorganic resources are distributed across the phylogeny.

Here, we measured species' abilities to compete for limiting resources to test whether an absence of phylogenetic signal of traits related to competition may explain previous findings that phylogenetic distance is a poor predictor of algal ecology. Specifically, we measured species’ minimum resource requirements for light, nitrogen and phosphorus. We also measured species’ elemental content and stoichiometry for carbon, nitrogen and phosphorus because these elemental ratios reflect species’ consumption vectors—or the ratios at which species remove these elements from the environment. Algae are known to compete for these inorganic resources [32], and the abilities of species to sequester and survive on limiting quantities of these resources have been used to correctly predict the outcome of competition for limiting resources in other algae and plants [25, 26, 33] using resource ratio theory [34]. Lastly, we measured two morphological traits because these traits have been hypothesized to be "master traits" that inherently control other traits related to competition due to allometric scaling relationships [35, 36], and because size confers a defense against grazing [36].

We used a variety of approaches to test whether phylogenetic distance is related to trait variation among species. First, we estimated the strength and significance of the phylogenetic signal of each trait across the phylogeny. Second, we tested whether phylogenetic distance (PD), measured as the sum of the branch lengths between species pairs [7, 9], was a good predictor of their trait differentiation. Third, we asked whether the data were more consistent with a null, Brownian Motion, model of evolution with other alternative phylogenetic models of trait evolution, or with a non-phylogenetic model of trait-variation. Model fitting cannot be used to infer the mechanisms of evolution (e.g. ecological speciation or adaptive radiation). However, it can be used to support or reject hypotheses about how evolution impacts trait variation over time [37]. Overall, we found algal traits related to competition showed less phylogenetic signal than expected based on a Brownian Motion null model of evolution. Phylogenetic distance was not a good predictor of trait variation, and model comparisons suggest that traits tended to be either randomly distributed with respect to the tree, or consistent with accelerating evolution towards the tips of the tree.

While these results are the first demonstration that a lack of phylogenetic signal may explain previous findings showing that phylogenetic distance among species does not predict species interactions, coexistence, co-occurrence or ecosystem functioning, we acknowledge that phylogenetic distance among species on a tree is not equivalent to the phylogenetic distance of species in a community. Further work must be done to demonstrate that these traits explain community assembly in order to confirm that this lack of phylogenetic signal explains previous findings at the community level.

## Materials and Methods

### Species selection and resource requirement experiments

We sought to measure traits for 51 of the 59 species on the phylogeny (Table A in S1 File). We selected these species because they were either a) among the 50 most frequently occurring species in lakes across North America, as described in the US EPA's National Lakes Assessment survey for 2007, or b) they occurred in our study lakes at the University of Notre Dame’s Environmental Research Center in the Upper Peninsula of Michigan [38]. This resulted in a list of 95 species which was then further restricted by our ability to c) obtain a culture of each species from culture collections, and d) to culture the species on a single common freshwater medium (COMBO [39]). These four conditions determined the species in our species pool.

We conducted resource-limitation experiments to determine how algal populations respond to gradients in resource availability, and to estimate species' minimum resource requirements (R*). We estimated species growth rates as a function of resource availability and used a Monod function to estimate the resource level at which the species could not grow (i.e. the zero net growth isocline, [34]). To do so, we performed three separate experiments in 48-well Falcon Tissue Culture Plates to describe the response of each species’ growth rate to increasing availabilities of nitrate, phosphate and light. Species were inoculated into sterile freshwater COMBO medium [39] and received 10% media exchanges every other day for 14 days. Well-plates were placed on a shaker table and rotated at 100 rpm and were illuminated on a 18L:6D photoperiod in an environmental chamber that was maintained at 20°C. We measured in situ fluorescence daily using a Biotek Synergy H1 Hybrid Reader, at an excitation wavelength of 435 nm and an emission wavelength of 685 nm to estimate in situ chlorophyll-a [40]. We used chlorophyll-a fluorescence as a proxy for algal biomass [41], and estimated species growth rates as a function of resource availability by using a Monod function to estimate the resource level at which the species could not grow (i.e. the zero net growth isocline, [34]).

We estimated growth rates (*r*) by fitting data on fluorescence values to the exponential time-series:

$$F_{t}=F_{0}e^{rt}$$ (1)

where F_t_ is the fluorescence on day t, and F_0_ is the fluorescence on day zero. We did not include time series that showed zero or negative growth in the analysis. Not all time series showed exponential growth dynamics for the entire time series. In many cases species displayed density-dependent growth by the end of the two weeks. In order to obtain the best possible estimate of the exponential portion of growth, we eliminated time points from the end of the time series until the variance explained by the exponential growth curve was maximized—as estimated by the R^2^ value of the curve. The population growth rates (r) were then compiled across the treatment levels for each species, with each species by resource concentration treatment being replicated twice.

We estimated each species' minimum light requirement and light-use efficiency by subjecting well-plates to 10 different levels of light and measuring each species’ population growth rate. The well-plates were illuminated under AgroBrite T5 fluorescent bulbs. We used neutral density filters (Solar Graphics Inc.) to reduce the total amount of irradiance that the algae receive in the well-plates, without changing the distribution of wavelengths. Light intensities under the filters were measured using a Biospherical Instruments QSL-2100 Radiometer, and were: 0, 1, 7.5, 10, 32.5, 48, 113.5, 133, 162.5, 198.5, 210.5, 237, 248.5, 311.5 μEinstein m^-2^ s^-2^.

In order to estimate algal growth requirements for nitrogen, we exposed each species to six increasing molar concentrations of nitrate in the form of NaNO_3_ (0, 0.2, 2, 20, 100, and 200 μmol L^-1^). For comparison, standard COMBO contains 1000 μmol L^-1^ of NaNO_3_, and the minimum and maximum molar concentrations of total nitrogen in the EPA National Lakes Assessment Survey of 1,157 lakes in 2007 were 0.07 µmol L^-1^ and 1,863 mol L^-1^, respectively. Nitrogen was provided solely in the form of NaNO_3_. All other nutrient concentrations were kept constant. These concentrations of nitrate were chosen based on a previously published resource limitation experiments on freshwater algae [26], as well as pilot studies in which we estimated minimum resource requirements on a small subset of our selected taxa across a wider gradient of resource availabilities.

Similarly, in order to estimate algal requirements for phosphorus, we employed six different molar concentrations of phosphate, in the form of K_2_HPO_4_ (0, 1, 2, 4, 8, and 16 μmol PO4 L^-1^). Standard COMBO contains 50 μmol L^-1^ of K_2_HPO_4_, and the minimum and maximum molar concentrations of total phosphorus in the EPA National Lakes Assessment Survey were 0.03 μmol L^-1^ and 157 μmol L^-1^, respectively. In phosphate removal treatments, we held the availability of potassium constant by replacing the same molar concentration of potassium in the form of KCl. All other nutrient concentrations were maintained as in standard COMBO medium. We soaked the well-plates and pipette tips used in the phosphorus experiment in a 10% HCl acid bath for 24-hrs to eliminate the possibility of phosphate contamination, after which they were rinsed in DI water. For both the nitrate and phosphate experiments the algae were exposed to 100 μEinstein m^-2^ s^-2^ of irradiance from Cool-White fluorescent bulbs. Well-plates were randomly assigned a location on the shaker table.

Prior to their inoculation into well-plates, species were grown in batch culture in COMBO medium. In order to remove existing nitrogen and phosphorus from the batch medium for the nitrogen and phosphorus experiments respectively, each culture was centrifuged at 4000 RPM for 15 minutes and rinsed with nitrogen- or phosphorus-free media three times. The algal pellet was then re-suspended in sterile COMBO medium. The algae were kept in nitrogen-free or phosphorus-free medium for the nitrogen and phosphorus experiments, respectively for 72 hours prior to the start of the growth measurements. This nutrient 'starvation-period' was employed to ensure that species would use up any remaining dissolved or stored nutrient resources in their cells before being exposed to the experimental nutrient treatments—i.e. to prevent nutrient contamination. Re-suspended cells were then counted under a compound microscope and either concentrated or diluted to a concentration of 6,000 cells mL^-1^. Each experimental well contained 900 μL of media and was inoculated with 100 μL of algae to achieve an initial density of 600 cells mL^-1^. The well-plate lids were sealed with Parafilm to prevent evaporative losses.

We used non-linear least squares regression in R (R Development Core Team 2011) to estimate the parameters α and μ_max_ of the Monod equation:

$$\mu \left(R\right)=\frac{\mu_{max}R}{R+\frac{\mu_{max}}{\alpha }}-D$$ (2)

where μ(R) is the population growth rate at a given resource concentration (R), α is the initial rate of response of the population growth rate to increases in the resource, μ_max_ indicates the maximal population growth rate in the condition of unlimited resources, and D is the dilution rate (0.05). The Monod equation is a saturating function that describes how the per capita population growth rate of a species increases with increasing resource availability. For the light experiment, we also fitted a second model which identifies an optimum light level (R_opt_), at which the population growth rate is maximized and above and below which the population growth rate declines [42]:

$$\mu \left(R\right)=\frac{\mu_{max}R}{\frac{\mu_{max}}{\alpha \;R_{opt}^{2}}R^{2}+\left(1-2\frac{\mu_{max}}{\alpha \;R_{opt}}\right)R\;+\frac{\mu_{max}}{\alpha }}-D$$ (3)

We used the AIC of the two model fits to select the best model. The Monod model was selected for all but three species (*Scenedsmus acuminatus*, *Selenastrum minutum* and *Spondylosium planum*). For these three species the light optimum model (3 above) provided the better fit. We then solved the parameterized growth functions in order to determine the level of each resource at which growth rates went to zero (N*, P* and I*) using Mathematica v. 9.0.1 (Wolfram).

#### Stoichiometry & Cell measurements

We estimated species' stoichiometric elemental ratios (e.g. N:P) because they reflect the homeostatic equilibrium of elements within each species' tissue—or the balance between what a species takes in and what it excretes [43, 44]. Stoichiometric ratios thus reflect a species' relative use of one element over another in carrying out its cellular functions, and they have been used as an estimate of species' resource consumption "vectors"—or the ratio at which a species is removing two resources from the environment [33]. These vectors, along with minimum resource requirements for two species, are the two pieces of information needed to predict the outcome of competition for two resources [34]. Furthermore, because certain elements are more important for cell growth (e.g. nitrogen) or cell division (e.g. phosphorus), stoichiometric ratios can also reflect variability in life history strategy among species, i.e. growing larger cells that divide slowly versus splitting into small cells that divide rapidly [43, 44]. Algal stoichiometry is known to display some plasticity (i.e. variation in response to environmental conditions) [36, 45], but intraspecific variation is generally lower that interspecific variation, and so species do generally have unique stoichiometric signatures [46]. To estimate the C, N, and P stoichiometry of the focal species, we centrifuged 300 mLs of batch culture to achieve a pellet of concentrated biomass of each species and poured off the supernatant. The pelleted biomass was dried in a drying oven at 60°C for 5 days. The dried algal biomass samples were then massed and sent to the University of California Davis Analytical Lab for determinations of nitrogen, phosphorus, and carbon content, and stoichiometry.

We estimated cell size and one aspect of morphology (long axis length), because size and morphology are considered to be "master traits" that are linked to competitive abilities and resistance to grazing for phytoplankton [47]. We measured cell dimensions for ten cells of each species using a camera attached to a compound light microscope to take photos and made size-calibrated measurements of each cell using Olympus' CellSens imaging software (Olympus Corporation of the Americas). Each species' cell shape was grouped according to standard shape-classifications a priori and cell dimensions were used to calculate the cellular biovolume of each cell (μm^3^) [48]. All trait data and meta-data are available online (S1 and S2 Tables respectively).

### Data analysis

In order to test whether ecologically relevant traits exhibit significant phylogenetic signal and to fit models of trait evolution, we relied on a recently published phylogenomic dataset for freshwater green algae [49]. The phylogeny, full methods and results for the phylogenomic analyses, as well as supplementary trees and raw data are available or referenced in [49]. All phylogenetic datasets and orthologs are available on Dryad (doi:10.5061/dryad.c574h). Raw Illumina files can be accessed using the NCBI SRA archive under BioProject Accession PRJNA237822. The bioinformatics tools can be accessed at http://galaxy-dev.cnsi.ucsb.edu/osiris, downloaded from https://bitbucket.org/osiris_phylogenetics/osiris_phylogenetics, and read about at http://osiris-phylogenetics.blogspot.com. To our knowledge, this is the most data-rich molecular phylogeny of freshwater green algae published to date.

For each trait, we chose to calculate three commonly used metrics of phylogenetic signal: Blomberg's K, Pagel's λ, and Moran's I using the Picante, Geiger and Adephylo packages in R respectively [50–52]. P-values for the significance of K and I were based on 999 randomizations each, whereas the significance test for λ was based on a likelihood ratio test. K and λ are considered to reflect an effect of shared ancestry on trait similarity, while I is considered to be a measure of autocorrelation of traits across the phylogeny. For K and λ, values close to zero indicate no phylogenetic signal, while values of one correspond to a Brownian motion expectation. Unlike λ, K can achieve values > 1, indicating that species are more similar than would be expected based on Brownian motion.

We tested for significant relationships between the pairwise phylogenetic distance among species (PD) and trait differentiation among species using Mantel tests, using the ‘ape’ package in R. PD was calculated using the ‘cophenetic’ function, and the trait distances were calculated as Euclidean distances using the ‘dist’ function. We considered trait differentiation and PD among species pairs to be significantly correlated at P≤0.05.

Most standard tests of phylogenetic signal assume that traits have evolved according to a Brownian Motion model [50, 51]. As a result, it is important to test whether or not a Brownian Motion model is indeed a good fit to the data, or if an alternative model provides a better fit. If alternative models provide a better fit, then metrics of phylogenetic signal based on a Brownian Motion model are not meaningful [17, 53]. Therefore, while some of the measured traits did display a phylogenetic signal, it was important to determine whether the Brownian Motion model of evolution was indeed a good explanation variation in these traits. Additionally, because many of our traits did not display a significant signal, we asked whether alternative models provided a better explanation of trait variation. For each trait, we tested nine models of continuous trait evolution using the Geiger package in R [54].

**Fig 1:**
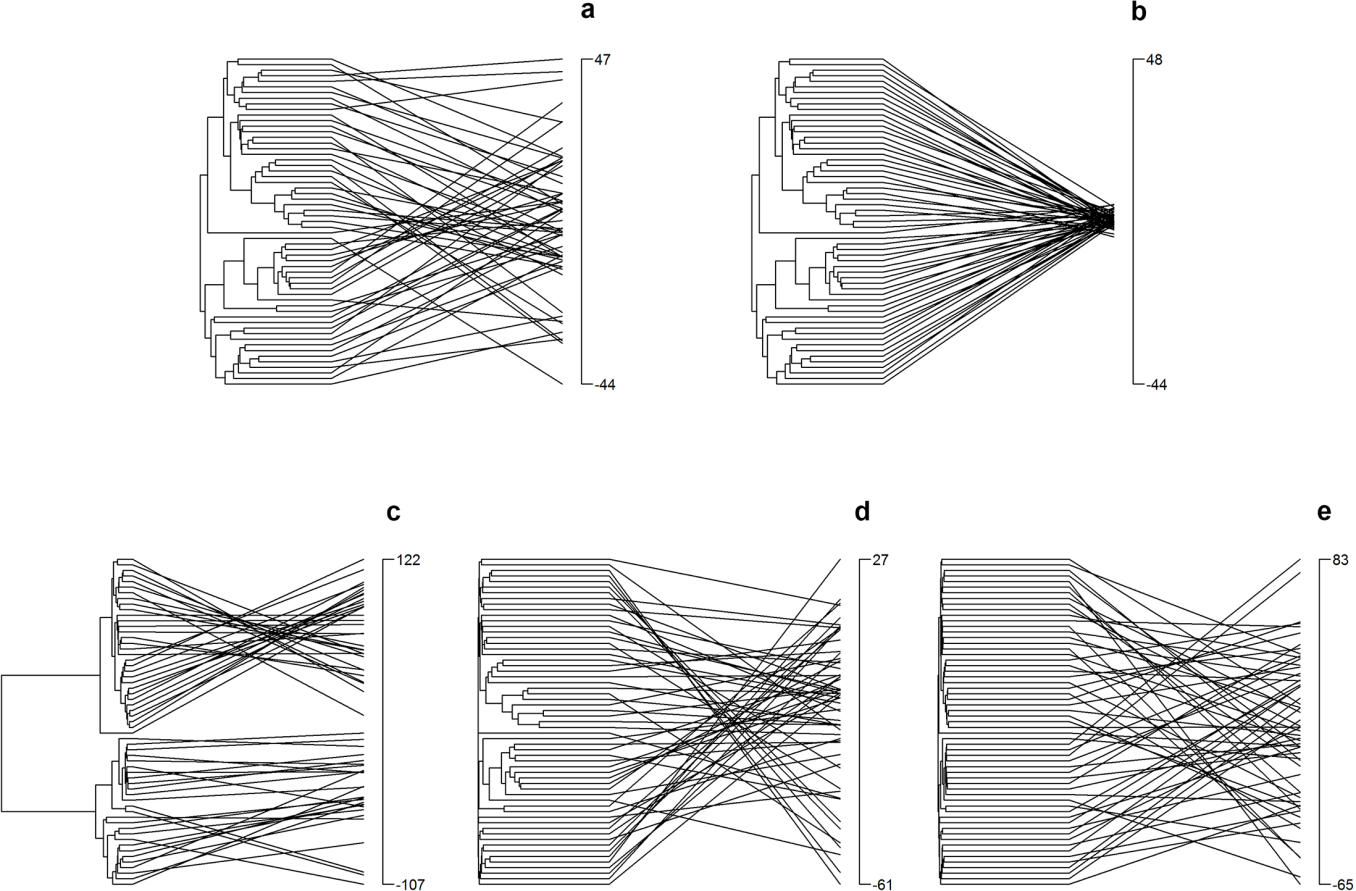
Simulated traitgrams indicating the relationship between each species' trait value (vertical axis on the right of each panel), and its phylogenetic position on the left for five different models of trait evolution: a) Brownian Motion (“BM”, root value = 0), b) Ornstein-Uhlenbeck (“OU”, θ = 1, α = 0.8), c) δ Model of a tree transformation with a low value of δ (0.1), d) δ Model of a tree transformation with a high value of δ (3), e) a λ Model of a tree transformation close to a star phylogeny, i.e. a low value of λ (0.1). We used the unsmoothed phylogenetic tree from Alexandrou et al. 2015 [49] for these simulations. The branches lengths of the tree were visually “smoothed” for the figures by extending a horizontal line between the tips of the phylogeny and the maximum tip value. Values are based on simulations and therefore have no units or biological meaning. The scale was held constant for Fig 1a and 1b to allow a comparison of the impact of the OU model on trait variation relative to the BM model. Scales for other models were allowed to vary. The function that was used to generate the simulated trait data is an update of the picante package for R called evolve.trait.R and is available on the R Forge website (R-Forge@R-project.org).

Five of the nine potential models of evolution in the Geiger package were retained for further analyses, either because they were the best fit model for one or more traits, or because they are useful for comparison with prior studies. The Brownian Motion (BM) model represents the evolution of trait as a random walk, and the model estimates a single rate parameter. The Ornstein-Uhlenbeck (OU) model aims to mimic the effect of stabilizing selection towards a single optimum and it therefore fits a model that describes the evolution of constrained trait variation. It incorporates a random trait walk, but includes two additional parameters, θ, which indicates the optimum trait value, and α, which represents the strength of the pull on trait values towards the optimum [55]. As the current trait value at any point in time deviates further from the optimum, the impact of α becomes proportionately larger. As a result, OU models will generally lead to constrained trait variation among species compared to a BM model (Fig 1B). The White Noise (WN) model is a non-phylogenetic model that assumes that trait data come from a random normal distribution, and that species have no significant trait covariance (hence not shown in Fig 1). The δ tree-transformation model transforms the phylogeny to reflect the relative importance of recent divergence versus shared ancestry in determining trait variability. The δ parameter represents the influence of accelerating or decelerating evolution; values < 1 indicate that stem branches contributed disproportionately to trait evolution, while values > 1 indicate that the tip branches contribute more heavily to trait variation [51]. For example, if distantly related species differ more than expected based on the phylogeny relative to recently diverged species, then it will transform the tree to shorten tip branches and lengthen stem branches, thereby lessening the impact of recent divergence on traits (δ < 1, Fig 1C). Conversely, if closely related species are more divergent than expected based on phylogeny, then it will transform the tree so as to lengthen the tip branches and exaggerate the impact of recent divergence (δ > 1, Fig 1D). The λ models also transform the tree so as to reflect the trait variability. However, λ only varies from 0 to 1, where λ = 1 retains the actual phylogeny and the traits are distributed across the tree as they would be for a BM model of evolution, and λ = 0 is a star phylogeny, where traits can be randomly dispersed across the phylogeny because common ancestry has no impact on trait variability (Fig 1E) [51]. To determine the relative likelihood of the five retained models, we calculated each model's Akaike's Information Criterion (AIC) and Akaike weight. All analyses were conducted using the published phylogram of freshwater green algae [49]. When no model achieved an AIC weight of 0.5 or more, we concluded that no model performed substantially better than the others (no choice).

## Results

Among the 17 traits that were measured, only three traits showed significant phylogenetic signal (measured as Blomberg's K): biovolume, long axis length and μ_max_ light (Table 1). There was also a marginally non-significant phylogenetic signal for %C (although K = 0.401 is lower than K = 1 expected for a Brownian Motion random walk). There was also some indication of a signal for μ_max_ phosphate (K = 0.857, p = 0.098), although it was not significant. There was no significant phylogenetic signal for traits related to resource requirements (R*s), responses to resource availability (αs), or stoichiometry (Table 1).

**Table 1:** Measures of evolutionary signal for freshwater algal ecological and physiological traits. K = Blomberg's K (p-value based on randomization), Pagel's λ (p-value based on log-likelihood ratio test with λ set to zero), Moran's I (p-value based on randomization and one-way hypothesis test). Biovolume and long axis length are measures of algal size and morphology respectively; α light, α nitrate and α phosphate are the initial rates of response of species' growth rates to increases in resource availability; μ_max_ light, μ_max_ nitrate and μ_max_ phosphate are the maximum per capita population growth rates of each species when that particular resource is unlimited; I*, N* and P* indicate concentrations of the resource (molar for N and P, μEinstein for I) at which the population growth rate equals zero; %C, %N, % P indicate the mass percent of carbon, nitrogen and phosphorus; C:N, C:P and N:P indicate the molar ratios of carbon (C), nitrogen (N) and phosphorus (P) of each species. N is the number of species.

Trait	K	p-value	λ	p-value	Moran's I	p-value	N
α light	0.425	0.209	**0.999**	**0.001**	-0.021	0.834	41
μ_max_ light	**0.394**	**0.011**	**0.832**	**0.064**	-0.040	0.607	40
I*	0.245	0.285	0.000	1.000	-0.034	0.743	41
α nitrate	0.302	0.191	0.110	0.703	-0.042	0.875	20
µ_max_ nitrate	0.057	0.694	0.000	1.000	-0.076	0.747	20
N*	0.426	0.124	0.000	1.000	0.007	0.304	20
α phosphate	0.454	0.426	0.000	1.000	-0.241	0.522	7
μ_max_ phosphate	0.857	0.098	1.000	0.140	-0.061	0.332	7
P*	0.587	0.356	0.000	1.000	-0.146	0.803	7
% C	**0.401**	**0.053**	**0.547**	**0.064**	-0.027	0.748	50
% N	0.169	0.476	0.292	0.197	0.011	0.137	50
% P	0.053	0.901	0.171	0.353	0.021	0.054	48
C:N	0.264	0.153	0.000	1.000	0.012	0.125	50
C:P	0.243	0.261	0.274	0.234	0.010	0.156	48
N:P	0.115	0.668	0.077	0.680	-0.029	0.722	48
Biovolume	**0.694**	**0.033**	**0.317**	**0.033**	-0.032	0.835	42
Long axis length	**0.661**	**0.037**	**0.292**	**0.064**	-0.031	0.677	42

The Mantel tests confirmed that the phylogeny provides some predictive value for trait variation for log biovolume and log long axis length (Table 2). There was a significant correlation between PD and the difference across species pairs in terms of their logged biovolumes and their logged long axis lengths. There was also a marginally non-significant correlation between PD and trait distance for the N:P and C:P molar ratios (Table 2). However, there was no significant relationship between PD and trait distance for any other traits related to competition. Altogether, this means that PD was a poor predictor of trait variation for 13 of the 17 traits that we measured.

**Table 2:** Results of Mantel tests to determine whether trait distances and PDs among pairs of algal species are correlated. The analysis was performed by comparing trait distance and PD matrices for each trait using the mantel.test function in the ‘ape’ package in R.

Trait	z statistic	p-value
α light	49.92	0.84
μ_max_ light	162.21	0.31
I*	313.38	0.70
α nitrate	326.87	0.41
μ_max_ nitrate	14.50	0.24
N*	2.14	0.22
α phosphate	94.40	0.21
μ_max_ phosphate	2.42	0.24
P*	0.86	0.45
% C	900.02	0.36
% N	2887.04	0.73
% P	122.70	0.30
C:N	2402.26	0.61
C:P	77739.05	0.07
N:P	2938.76	0.08
**log biovolume**	436.10	**0.001**
**log long axis length**	189.63	**0.001**

**Fig 2:**
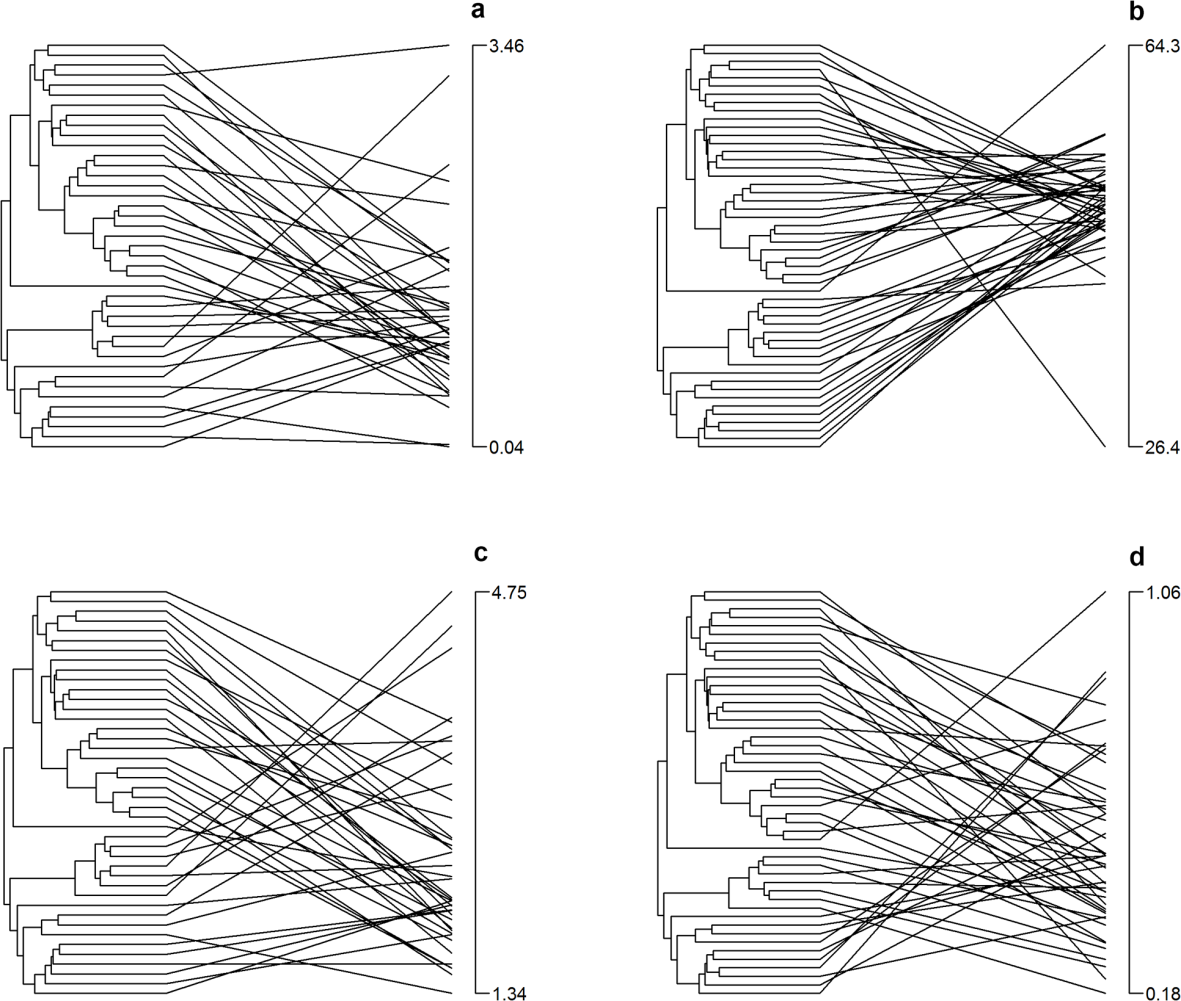
Traitgrams indicating the relationship between each species' trait value (vertical axis on the right of each panel), and its phylogenetic position on the left. **a)** Distribution of I* across the phylogeny. For this trait a WN model was supported, indicating a random distribution of traits across the phylogeny; **b)** Distribution of % C across the phylogeny, a WN model was supported, again indicating a random trait distribution; **c)** Distribution of log-transformed algal biovolume across the phylogeny, a δ model was supported with a δ value of 7.971, suggesting a very weak influence of ancestral branches on trait variation; and **d)** Distribution of % P across the phylogeny, a λ model was supported with a λ value of 0.171, indicating that a tree-transformation to a nearly star-like phylogeny was the best fit. The phylogeny in this figure is the phylogeny published in Alexandrou et al. 2015 [49]. We have smoothed the branches of the phylogeny only for purposes of the illustration.

The model selections indicated that the WN model was the most commonly supported model. The WN model provided the best fit to the data for 7 out of 17 of the traits, including μ_max_ light, I* (Fig 2A), α phosphate, μ_max_ nitrate, % C (Fig 2B), % N, and the C:N molar ratio (Table 3). Phylogenetic models of trait evolution (e.g. δ and λ) were also supported for 7 traits. The δ model received the greatest amount of support for species' biovolume (Fig 2C), long axis length, α nitrate and N* (Table 3). For all of these traits, the δ parameter was > 3, suggesting that evolution of these traits occurred predominantly in the tips of the tree. This means that species are more different from one another than expected based on the extent of their shared ancestry and a Brownian Motion model of evolution. The λ model received the greatest support for %P (Fig 2D), C:P molar ratio and N:P molar ratio, although the WN model also received low to moderate support for all three traits. The λ parameter was close zero for these traits, indicating that a tree-transformation to a star phylogeny provided the best fit to the data (i.e. evolutionary relatedness was not correlated with trait similarities among species). For three traits, no model received strong support (AIC weights were all <0.50), namely, α light, μ_max_ phosphate and P*. For the two phosphorus traits, μ_max_ phosphate and P*, this lack of model support may have been due to the limited sample size and the resulting low statistical power for these traits (N = 7 species), which occurred because many species failed to grow under the experimental conditions after the imposed 72-hour nutrient starvation.

**Table 3:** Trait of interest, best model, Akaike (AIC) weights and parameter estimates for models of trait evolution. BM = Brownian Motion model, OU = Ornstein-Uhlenbeck model, WN = White Noise model. Models were fitted to the trait data using the Geiger package and the fitcontinuous function in R. Algal traits are the same as in **Table 1**. See Fig 2 for traitgrams. The α parameter of the OU model, the δ parameter of the δ model and the λ parameter of the λ model are all also shown to allow interpretation of the model selections. In cases where a δ Model or a λ model received the greatest support from AIC weights, we tested whether the estimated parameter value was significantly different from a BM expectation by simulating 1,000 random walks of evolution. For each simulated walk, we then estimated δ and λ, creating sampling distributions of the parameters to determine the estimated parameter value from our data was outside of the 95% confidence interval for a random walk. Parameter values that are in bold are outside of the 95% confidence interval for a random walk of evolution (α CI = 0.00–3.16 (one-tailed), δ CI = 0.52–4.00 (two-tailed), λ = 0.87–1.00 (one-tailed)).

Trait	Best model	WN	BM	OU	δ model	λ model	α parameter	δ parameter	λ parameter
α light	no choice	0.125	0.222	0.381	0.189	0.083	7.812	0.000	0.999
µ_max_ light	**WN**	**0.539**	0.029	0.132	0.052	0.248	12.139	0.000	0.832
I*	**WN**	**0.957**	0.000	0.000	0.000	0.043	60.651	2.121	0.000
α nitrate	**δ**	0.066	0.001	0.005	**0.672**	0.255	25.424	3.617	0.110
µ_max_ nitrate	**WN**	**0.689**	0.000	0.000	0.000	0.311	1000.000	4.857	0.000
N*	**δ**	0.029	0.014	0.013	**0.895**	0.048	13.522	4.879	0.000
α phosphate	**WN**	**0.560**	0.092	0.077	0.077	0.194	20.454	2.440	0.000
µ_max_ phosphate	no choice	0.261	0.313	0.172	0.139	0.115	4.698	0.579	1.000
P*	no choice	0.391	0.166	0.098	0.171	0.174	13.574	7.207	0.000
% C	**WN**	**0.937**	0.003	0.034	0.001	0.024	17.317	0.004	0.547
% N	**WN**	**0.507**	0.000	0.000	0.000	0.493	300.572	3.386	0.292
% P	**λ**	0.476	0.000	0.000	0.000	**0.524**	805.374	4.557	**0.171**
C:N	**WN**	**0.903**	0.000	0.000	0.000	0.097	61.044	2.131	0.000
C:P	**λ**	0.297	0.000	0.000	0.001	**0.702**	85.500	0.000	**0.274**
N:P	**λ**	0.385	0.000	0.000	0.000	**0.615**	732.866	4.211	**0.077**
Biovolume	**δ**	0.000	0.000	0.000	**1.000**	0.000	0.000	7.888	0.317
Long axis length	**δ**	0.000	0.000	0.000	**1.000**	0.000	0.000	4.613	0.292

Despite the fact that Blomberg's K and Pagel's λ values indicate that there was significant phylogenetic signal in μ_max_ light, %C, biovolume and long-axis length (Table 1), both the K and λ values tended to be less than one, indicating that the strength of the signal was weaker than would be expected based on a BM model of evolution. Furthermore, the model selections identified the WN model to be the best model for μ_max_ light and %C and the δ model as the best model for both biovolume and long axis length, each with δ parameters of >4, suggesting that common ancestry does not heavily constrain variation in cell size and morphology among species. For biovolume and long axis length, the support for the δ model was strong (~1), and there was no support for the BM model. Blomberg's K values can be misleading when Brownian Motion is not a good underlying model of evolution [17, 53], and as a result, we do not interpret the significant K statistics as robust evidence of phylogenetic signal in these traits. However, the Mantel tests confirmed that there is a significant positive correlation between PD and trait distance among pairs of species for log biovolume and log long axis length, and so, we conclude that while phylogenetic distance does provide some information about differences in biovolume and long axis length among species, the correlation is weaker than expected based on a neutral model of trait evolution.

The evolutionary model selections and parameter values generally indicate that species' phylogenetic placements on the tree are not strongly related with their trait values. In some cases a non-phylogenetic model (WN) was selected, while for other traits, phylogenetic models (δ and λ) provided the best fit. The WN model indicates that traits were randomly distributed across the tree, while the model parameters for the δ and λ models indicated that the stem branches, or shared ancestry, had little influence on trait variation. These various model selections therefore lead to the similar interpretations. Using simulation, we show that traits that are randomly distributed across the tree, which are expected to return a WN model as the best fit model, also sometimes return δ and λ models (with parameter values reducing the influence of stem branches) as the best fit model (see Supporting Information). For traits that are randomly distributed across the phylogeny, which model is selected may have more to do with the statistical trait distribution than the association of the traits with the phylogeny. Regardless of the identity of the model selected, for 14 model selections, the biological interpretation of the model and parameter estimates selected is essentially very similar: shared ancestry has little ability to predict trait similarity.

## Discussion

### Major findings

Overall, our data do not lend much support to the hypothesis that ecological traits related to competition in freshwater green algae (resource requirements, resource assimilation, stoichiometry) are related to the phylogenetic placements of species on the tree of life. We found that for 13 of 17 traits, there was no significant phylogenetic signal. We also used a model-fitting approach to test whether the data supported a Brownian Motion model of evolution, a model of constrained evolution (OU), models of accelerating or decelerating evolution (δ and λ), or a White Noise (WN) model of essentially random trait distributions. The AIC weights indicated that a White Noise (WN), non-phylogenetic, model of evolution was most frequently supported. Even when the Blomberg’s K values indicated a significant phylogenetic signal, non-phylogenetic models were selected, or the best phylogenetic models indicated that traits have diverged since the most recent speciation events. Specifically, in the instances where phylogenetic models were supported (i.e. δ and λ), the estimated parameter values indicate that the phylogenetic signal was weak (K<1), evolution was accelerated in the tips of the phylogeny (δ ≥ 3), or that a star phylogeny transformation was the best fit (λ close to zero). Taken collectively, these model fits and parameter estimates suggest that ecological traits were not closely related to patterns of common ancestry (low signal), and that species' trait values often varied randomly across the tree (frequent selection of white noise as the best model). While other studies have shown that phylogenetic signal may not occur for all traits [19, 56–60], these results are novel because, to our knowledge, this is the first test of signal in traits related to resource competition in freshwater green algae. These results contrast with prior studies indicating that body size variation in diatoms evolved in basal lineages [61], and that variation in carbon uptake ability among groups of algae and other microbes occurs across larger taxonomic groups [62–64]. Similar to our findings however, research on prokaryotic microorganisms has also shown that variation in resource use, specifically in nitrate and phosphate uptake affinities, does not display a phylogenetic signal due to recent divergence [62, 65, 66].

The lack of phylogenetic signal in most of the ecological traits suggests that evolutionary relatedness does not generally reflect biological variation that is thought to underlie competitive abilities and resource use differentiation among freshwater green algae. While this contrasts with the expectation that more closely related species should be more similar in their traits than distantly related species, the absence of phylogenetic signal in species' traits is not uncommon in other taxa [19, 50]. Furthermore, these results may help to explain previous findings which demonstrated that the phylogenetic distance among pairs of freshwater green algal species is not a good predictor of the size of niche or fitness differences between them [3], the strength of their species interactions [31], or the level of ecosystem functioning they produce [10]. These results may also provide an explanation for the observed lack of phylogenetic structure in 99% of the >1,000 natural communities of freshwater algae that have been surveyed in North American lakes [49]. Specifically, if competition for resources is important in structuring natural communities [67], but competitive traits do not display a phylogenetic signal, then we should not expect natural communities to display phylogenetic structure [15].

### Biological reasons for the absence of phylogenetic signal

There are a number of potential biological explanations for the lack of a relationship between species' placements on a molecular phylogeny and the trait values, as overwhelmingly observed in our dataset. While we cannot assess these directly with the data collected in this study, they are worth discussing in order to provide guidance for future work. First, it is possible that trait plasticity (variation of trait values within a species that occurs in response to variability in the environment) is obscuring phylogenetic signal. Some of the traits that we measured, e.g. light utilization traits, are known to be plastic [36, 45, 68]. When variation among individuals in a species is sufficiently large to approach variation between species, any correlation between species traits and their evolutionary relatedness can be masked. However, for many of our traits (e.g. stoichiometry and nutrient utilization traits), previous work has shown that interspecific trait variation is greater than intraspecific trait variation despite plasticity, such that species have unique trait signatures [36, 46]. As long as interspecific trait variation is greater than intraspecific trait variation, plasticity should not limit the detection of phylogenetic signal.

There is also evidence that algal traits can evolve rapidly, i.e. so quickly that they can affect the impact of ecological interactions as they are occurring [69–72]. Although this has only been demonstrated for traits related to herbivore defense, it is possible that the lack of phylogenetic signal that we found in competitive traits may be due to high trait lability. The individual functional genes controlling those traits may also be evolving at much faster rates than the rest of the genome. The phylogeny used in this study is based on many genes with the intention that using as many genes as possible will produce a phylogeny that best reflects the species tree. However, the genome is inevitably composed of genes that evolve at different rates, and individual gene trees may support alternative topologies [73]. If the genes responsible for a particular ecological function are evolving at a different rate than the majority of the genome, or with a different pattern of descent, then ecological function would be decoupled from overall genetic sequence divergence, and therefore phylogenetic distance.

Lastly, we know very little about the relationship between genotype and phenotype evolution for algal species. For example, while many nucleotide substitutions are functionally synonymous, some do result in changes of large effect, e.g. [74]. The functionality of any particular gene may also change independently of its sequence divergence via a number of mechanisms including changes in gene expression, methylation, RNA editing, or post-translational modification, among others. Thus, very different sequences can yield similar functions but large estimates of PD, while similar sequences with only a few unique base pairs may yield small estimates of PD but have vastly different functionality. Additionally, the traits we measured may be determined by multiple genes that interact to produce phenotypes in a non-additive or non-linear fashion (epistasis) [75, 76]. Future work should focus on better characterizing and incorporating these nonlinear impacts of genetic variation on phenotype into models of trait evolution. Gene knock-out or knock-down experiments could be used to identify and describe the way in which genes, sequence variability, and gene interactions control traits related to competition.

### Study limitations

There are some important limitations to this study. First, while we are confident that the traits we measured do reflect species' abilities to acquire and survive on limiting resources, we did not directly assess the ability of these traits to predict the outcome of resource competition for these species. In order to confirm that the absence of a phylogenetic signal in these traits indeed explains our earlier findings that phylogenetic distances among freshwater green algae do not predict the strength of competition, or more generally, species interactions, community structure and ecosystem functioning, we would need to test whether these traits correctly predict these aspects of community ecology. Most studies of phylogenetic signal however, measure physiological, morphological and life history traits that are thought to be only indirectly linked to resource acquisition and the outcome of competition (e.g. [77, 78]). Others measure some aspect of resource use, such as diet or habitat preferences [19]. Such traits may be the best possible, though indirect, estimates of resource requirements and competitive abilities for many taxa. The traits that we measured, however, are direct estimates of resource requirements and competitive abilities for inorganic resources, and have previously been shown to predict the outcome of competition for phytoplankton [25, 26, 79–81].

Secondly, some have argued that because phylogenetic signal in trait variation must occur at some phylogenetic scale [18], an absence of phylogenetic signal suggests that one has not studied the right phylogenetic scale [19, 82, 83]. For example, phylogenetic signal in the traits that we studied here may occur at smaller (e.g. among sister taxa, [18]) or larger phylogenetic scales (e.g. when comparing groups such as diatoms, coccolithophorids, dinoflagellates and green algae, [84]). Our goal here, however, was not to find the phylogenetic scale at which these traits display a phylogenetic signal. Rather our goal was to test whether common ancestry can be used to describe the variation in traits that reflect resource competition in freshwater green algae *per se*, because we had previously shown that these algae display great variation in the outcome of competition [3, 10, 31]. It remains possible however that these traits do display phylogenetic signal at other phylogenetic scales.

Finally, our species selections and methods may have impacted our conclusions because the species that grew in these experiments are only a subset of those found in nature. Specifically, we only included cultivable species that are common in lakes across North America and we excluded species whose traits we could not measure. Efforts to measure traits *in situ* and sample a greater species pool would help to test the generality of our findings.

In conclusion, our study demonstrates that phylogeny is not a good predictor of important ecological characters in freshwater green algae, and that maximizing phylogenetic distance among species is no more likely to capture diversity of some traits than randomly selecting species. While this is the first time this has been shown for competitive traits of freshwater green algae, a lack of phylogenetic signal has also been described in other traits and other taxa [19, 21, 56–60]. This suggests that tests of phylogenetic signal and evolutionary model fits of ecologically important characters should be regularly completed before using phylogenetic information to estimate phenotypic or functional biodiversity, to infer processes of community assembly, or to gauge ecosystem functioning.

## Supporting Information

S1 File (DOCX)

S1 Table Original trait data file. (TXT)

S2 Table Metadata file for trait data. (TXT)
