# Supplementary material for: Common Ancestry Is a Poor Predictor of Competitive Traits in Freshwater Green Algae
Source: PLoS One. 2015 Sep 8;10(9):e0137085. doi: 10.1371/journal.pone.0137085 (PMC4562640; doi:10.1371/journal.pone.0137085)
Supplement: S1 File — (DOCX) [file pone.0137085.s001.docx]

## Supporting Information

**Table A.** The list of 51 species used in the trait experiments. The culture collection names are abbreviations. UTEX = The University of Texas at Austin (USA), SAG = Sammlung von Algenkulturen Göttingen (Göttingen, Germany), CPCC = the Canadian Phycological Culture Centre (Ontario, Canada), CCAP = the Culture Collection of Algae and Protozoa (Oban, Scotland).

| Species | Culture Collection | Strain Number |
| --- | --- | --- |
| *Actinastrum hantzschii* | UTEX | LB 605 |
| *Ankistrodesmus falcatus* | UTEX | B 749 |
| *Arthrodesmus convergens* | SAG | 128.8 |
| *Botryococcus braunii* | UTEX | 2441 |
| *Botryococcus sudeticus* | UTEX | 2629 |
| *Chlamydocapsa ampla* | SAG | 122.8 |
| *Chlamydomonas moewusii* | UTEX | 2020 |
| *Chlamydomonas reinhardtii* | SAG | 81.72 |
| *Chlorella sorokiniana* | UTEX | 2805 |
| *Chlorococcum aquaticum* | UTEX | 2222 |
| *Closteriopsis acicularis* | SAG | 11.86 |
| *Closterium acerosum* | UTEX | LB 1075 |
| *Coelastrum cambricum* | UTEX | 2446 |
| *Coelastrum microporum* | UTEX | B 280 |
| *Coelastrum reticulatum* | UTEX | LB 1365 |
| *Cosmarium botrytis* | UTEX | B 175 |
| *Cosmarium turpinii* | UTEX | LB 733 |
| *Crucigenia tetrapedia* | SAG | 218-3 |
| *Dictyosphaerium ehrenbergianum* | CCAP | 222/1A |
| *Dictyosphaerium pulchellum* | SAG | 222-2a |
| *Elakatothrix viridis* | SAG | 47.96 |
| *Gloeocystis gigas* | UTEX | LB 291 |
| *Gloeomonas kupfferi* | UTEX | LB 603 |
| *Golenkinia minutissima* | UTEX | 929 |
| *Micractinium pusillum* | SAG | 13.81 |
| *Monoraphidium minutum* | UTEX | 2459 |
| *Monoraphidium arcuatum* | SAG | 14.81 |
| *Mougeotia sp.* | UTEX | LB 758 |
| *Oocystis parva* | SAG | 82.8 |
| *Oocystis polymorpha* | UTEX | 1645 |
| *Oocystis solitaria* | SAG | 83.8 |
| *Pandorina charkowiensis* | UTEX | LB 840 |
| *Pediastrum boryanum* | SAG | 261-7 |
| *Pediastrum duplex* | UTEX | LB 1364 |
| *Pediastrum tetras* | UTEX | B 84 |
| *Planktosphaeria botryoides* | UTEX | LB 951 |
| *Quadrigula closterioides* | SAG | 12.94 |
| *Radiococcus polycoccus* | SAG | 2361 |
| *Scenedesmus acuminatus* | SAG | 38.81 |
| *Scenedesmus ecornis* | SAG | 2332 |
| *Scenedesmus obliquus* | SAG | 276-3a |
| *Scenedesmus quadricauda* | UTEX | LB 614 |
| *Selenastrum capricornutum* | UTEX | 1648 |
| *Sphaerocystis schroeteri* | SAG | 16.84 |
| *Spondylosium planum* | SAG | 41.81 |
| *Staurastrum cingulum* | SAG | 40.83 |
| *Staurastrum punctulatum* | SAG | 679-1 |
| *Staurastrum tetracerum* | SAG | 7.94 |
| *Tetraedron minutum* | UTEX | LB 1367 |
| *Tetrastrum heteracantum* | UTEX | 2445 |
| *Volvox tertius* | UTEX | LB 132 |

**Randomization tests of model-fitting procedures**

The model fits for the green algal traits that we measured suggest a similar lack of phylogenetic control across the traits. Given this lack of signal, and the similar conclusions drawn from the various model selections and fits, it was not apparent why one model was selected over another in any particular case. We therefore sought to test the ability of the model fitting procedure to correctly distinguish different models of evolution. We simulated trait evolution by Brownian motion along our phylogeny to create a distribution of traits under our null expectations of a random walk (using David Ackerly’s function ‘evolve.trait’, available on R-forge, www.r-forge.r-project.org). To remove phylogenetic structure, we then randomly shuffled the data along the tips and tested the ability of the model-fitting procedure to correctly recover a White Noise model. The model fitting procedure recovered the non-phylogenetic White Noise model for the randomized data 95% of the time for 100 replicates (Fig. S1).

We then used our trait data and phylogeny to conduct the same test and to compare our model fit frequencies to that generated from simulated data, with the expectation that the frequencies should not be different from those generated from simulated, non-phylogenetic trait data (above). We randomized each trait 100 times along the phylogeny and recalculated the degree of phylogenetic signal and the best model fit. For 9 out of 16 traits, the distribution of model fits was significantly different from the randomized simulated Brownian motion trait (Table S2, Fig. S1). This randomization test returned parameters similar to the model fits for the real data: λ values were on average very low, indicating a star phylogeny was preferred, and δ values were typically high (though bimodally distributed), also indicating little phylogenetic signal. This implies that the choice of λ or δ over white noise may simply be an artifact of the data structure, instead of demonstrating an association of the traits across the tree. Understanding how trait distributions can impact model selection is beyond the scope of this work, but bears further investigation.

**Table B**. Model fits for trait randomizations. Percentages indicate what percentage of model fits selected the White Noise, λ, or δ models. Mean λ indicates the mean parameter estimates for model selections that selected the λ model. The Max and the Min δ indicate the range of δ values returned my model selections for the δ model. The χ^2^ test statistic and p-value estimates whether the distributions of model selections are significantly different from random.

|  | % White noise | % λ model | % δ model | Mean  λ parameter | Max.  δ  parameter | Min.  δ parameter | χ^2^ | P value |
| --- | --- | --- | --- | --- | --- | --- | --- | --- |
| α light | 48 | 8 | 44 | 0 | 3.00 | 0.02 | 60.14 | **8.73E-14** |
| α nitrate | 65 | 24 | 11 | 0.12 | 3.00 | 0.49 | 29.07 | **4.86E-07** |
| α phosphate | 85 | 15 | 0 | 1 | NA | NA | 4.5 | **0.033** |
| C:N | 90 | 10 | 0 | 0.09 | NA | NA | 1.15 | 0.28 |
| C:P | 89 | 10 | 1 | 0.18 | 2.23 | 2.23 | 2.86 | 0.24 |
| I* | 83 | 15 | 2 | 0.1 | 1.95 | 1.45 | 7.81 | **0.020** |
| Long axis | 48 | 17 | 35 | 0 | 3.00 | 0.04 | 56.99 | **4.21E-13** |
| N:P | 87 | 12 | 1 | 0 | 0.50 | 0.50 | 4.23 | 0.12 |
| N* | 61 | 28 | 11 | 0.14 | 3.00 | 0.15 | 34.44 | **3.32E-08** |
| %C | 61 | 29 | 10 | 0.07 | 3.00 | 0.16 | 34.35 | **3.47E-08** |
| %N | 90 | 9 | 1 | 0.08 | 0.88 | 0.88 | 2.28 | 0.32 |
| %P | 85 | 15 | 0 | 0.23 | NA | NA | 4.5 | **0.03** |
| P* | 100 | 0 | 0 | NA | NA | NA | 3.28 | 0.07 |
| µ_max_ light | 65 | 33 | 2 | 0.01 | 3.00 | 0.30 | 28.26 | **7.31E-07** |
| µ_max_ nitrate | 90 | 8 | 2 | 0.2 | 3.00 | 1.43 | 2.83 | 0.24 |
| µ_max_ phosphate | 95 | 5 | 0 | 0.89 | NA | NA | 0 | 1 |

**Fig A.** Model fits for all trait data, randomized and fit to 3 models of evolution 1000 times. The bottom bar indicates model fits for a trait simulated via Brownian motion and then randomly shuffled across the tips. * = χ^2^ test significant at the 0.05 level, *** = significant the 0.001 level.
